# Mixed Epithelial Stromal Tumor of the Kidney With a Mesenteric Lymph Node: A Case Report and Literature Review

**DOI:** 10.7759/cureus.46058

**Published:** 2023-09-27

**Authors:** Chitramalya Dan, Akshat Sahai, Priyasha Suri, Jaideep Singh, Ram S Trehan

**Affiliations:** 1 Hematology and Oncology, Greater Washington Oncology Associates, Silver Spring, USA

**Keywords:** endosalpingiosis, renal neoplasm, hormonal imbalance, mest, kidney

## Abstract

Mixed epithelial and stromal tumor (MEST) of the kidney belongs to the broad spectrum of renal neoplasms, distinguished by their varying composition of stromal to epithelial components. The histopathological display of the biphasic growth pattern of mesenchymal and epithelial elements, often with estrogen and progesterone receptor positivity, clinches the diagnosis. It is typically benign, with low recurrence rates and excellent prognosis after surgical resection. MEST constitutes a rare and unique subset, with limited research and understanding, requiring differentiation from other renal tumors. Our patient's presentation of a morphologically benign renal MEST with an imaging-positive inferior mesenteric lymph node renders this case exceptionally rare.

## Introduction

Within the diverse spectrum of renal tumors featuring a wide range of aggressiveness and malignant potential, mixed epithelial and stromal tumor (MEST) stands out as a unique entity. Developing from Müllerian-like stromal cells, MEST represents a rare kidney neoplasm, accounting for a mere 0.2% of renal cancers [1]. It was first recognised and reported by Michal and Syrucek in 1998 and subsequently bestowed a distinct position within the confines of the World Health Organization classification of renal tumors in 2004 [1,2]. Most commonly reported in perimenopausal and postmenopausal women, MEST has a noteworthy 1:10 male-to-female ratio. This has been attributed to hormonal imbalance or the use of hormone replacement therapy, even in the cases reported in males [1,3]. These tumors present a complex biphasic cystic-solid composition, mimicking ovarian stromal tissue and epithelium-lined cyst structure, requiring partial or radical nephrectomy, due to challenges in clinching the diagnosis solely by radiological assessment and being the preferred treatment modality [3,4]. In general, renal MEST is considered to have an excellent prognosis after surgical excision of the tumor; however, some sporadic reports exist of recurrence or malignant transformation [5]. In this report, we present a case of MEST with an imaging-positive mesenteric lymph node, managed surgically and proven benign on histopathology, providing intricate clinical, pathological, and radiological insights.

## Case presentation

**Figure 1:**
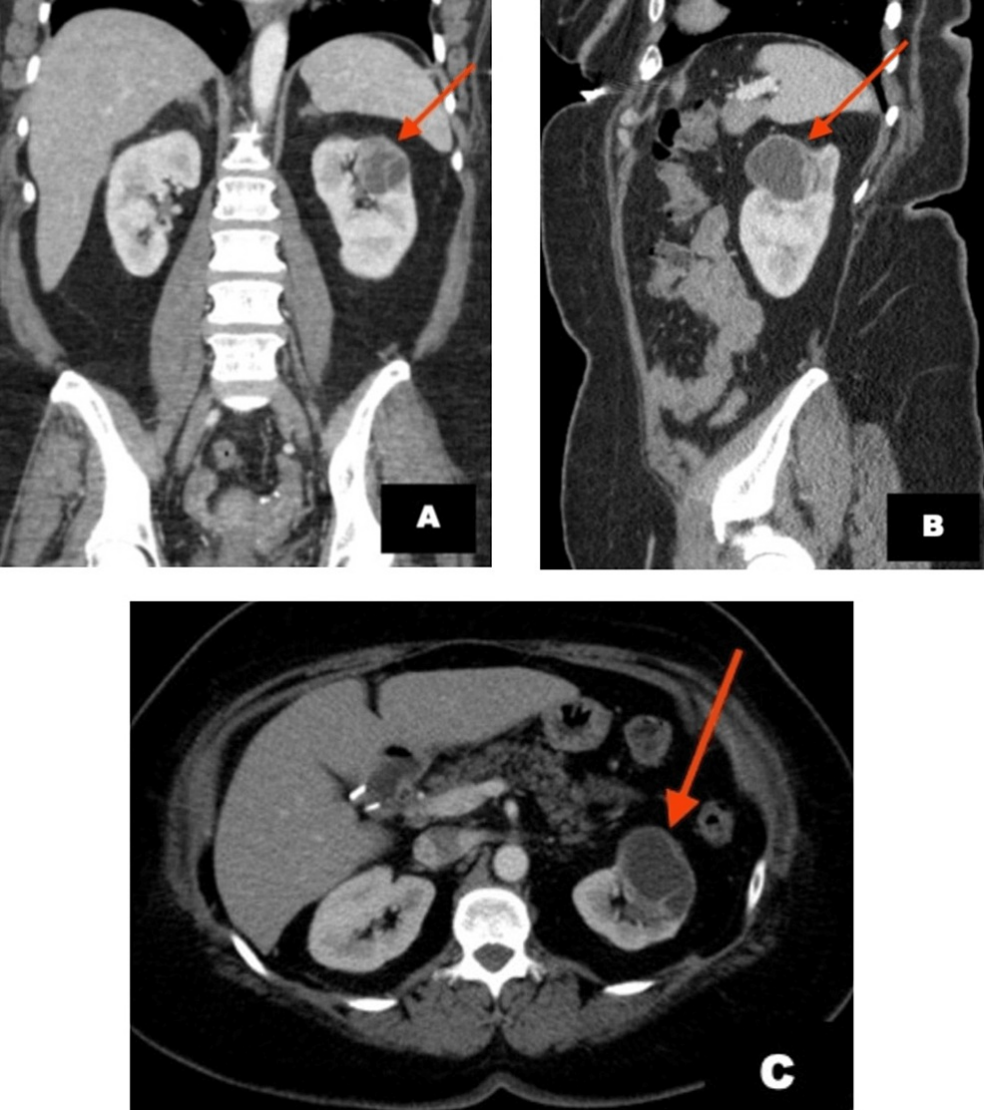
Lesion as observed on contrast-enhanced computed tomography scanning: (A) coronal view, (B) sagittal view, (C) axial view The study confirmed the presence of a partially exophytic, hypodense, cystic lesion in the upper pole of the left kidney with internal septations and no obvious perilesional fat stranding or edema.

A 60-year-old female presented with a history of low-grade, left-sided backache of three-month duration and recent onset gross hematuria with no complaints of weight loss, nausea or vomiting. She reported no personal or family history of renal calculi. She had been postmenopausal for almost 10 years and had a remote history of having undergone cholecystectomy, appendectomy, endometrial ablation and salpingectomy. She had a history of impaired glucose tolerance, hyperlipidemia and morbid obesity (BMI 38.4 kg/m^2^) managed with diet and exercise. Her family history was significant with the diagnosis of breast cancer in her maternal grandmother and alcohol-induced cirrhosis in her mother. On investigation, her urinalysis showed crystals; remaining urinary parameters and cytology were normal. Renal ultrasound (US) revealed a non-specific complex left renal cyst measuring 5.7 x 5.4 x 3.9 cm with hypoechoic internal contents and preserved renal cortical thickness. Contrast-enhanced computed tomography (CT) scanning of the abdomen and pelvis confirmed a 3.8 x 4.1 cm lesion at the upper pole of the left kidney exhibiting partial contrast-filling during excretory phases compatible with large calyceal diverticulum. Septation was noted at the lateral margin of this lesion. Cystoscopy, done in view of gross hematuria, was normal. Subsequently, she was lost to follow-up. She presented a year and a half later, with a recurrence of symptoms. A follow-up contrast-enhanced CT scan confirmed the previously described calyceal diverticulum in the upper pole region of the left kidney, measuring approximately 3.3 x 3.6 x 2.9 cm, with septation within (Figure 1). A novel finding was that of eccentric wall thickening of the calyceal diverticulum. An enlarged inferior mesenteric lymph node measuring 2.1 cm located within the mesentery anterior to the bifurcation of the common iliac arteries was noted. No invasion was noted in adrenal glands, and the head, body, and tail of the pancreas. Patency of the portal vein, celiac artery, superior mesenteric artery, renal vessels, inferior mesenteric artery and inferior vena cava was established.

**Figure 2:**
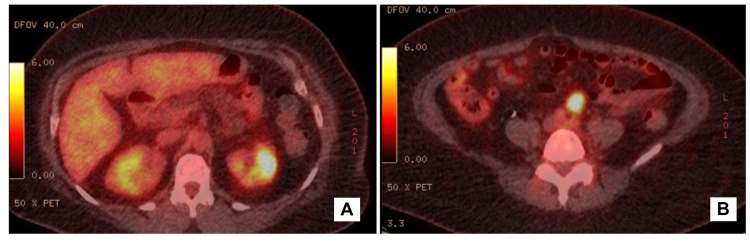
Lesion as observed on the skull base to mid-thigh FDG-PET scanning before the surgical intervention (A) Hypermetabolic lesion in the upper pole of the left kidney. (B) Hypermetabolic inferior mesenteric lymph node FDG-PET, fluorodeoxyglucose-positron emission tomography

These findings warranted a fluorodeoxyglucose-positron emission tomography (FDG-PET) scan. The FDG-PET scan noted the lesion in the upper pole of the left kidney to be hypermetabolic with a maximum standardized uptake value (SUV) of 9.9 and the lymph node anterior to the aortic bifurcation to be hypermetabolic with a maximum SUV of 12.0 (Figure 2). No abnormal activity was noted in the liver, retroperitoneum or in the pelvis. For a definitive diagnosis, a CT-guided, percutaneous fine-needle aspiration (FNA) of the left renal mass was done after obtaining patient consent. The procedure was tolerated well with no complications. However, microscopic examination reported the samples to be virtually acellular with red blood cells and adjudged inadequate for interpretation. Since the fine-needle aspiration cytology (FNAC) did not provide adequate information, the patient was recommended surgery. Written consent was obtained and the patient was thoroughly investigated before undergoing left robotic-assisted laparoscopic radical nephrectomy and lymph node dissection. The entire left kidney was excised along with the surrounding fat, upper part of the left ureter, and adjacent lymph nodes. While dissection, hilar vessels and regional structures were found to be free of the tumor. The patient tolerated the procedure very well and was discharged on the second postoperative day.

On pathological examination, the left kidney proper measured 11.5 x 5.5 x 4.5 cm and weighed approximately 200 grams. It was tan-yellow in appearance with lobulated perinephric fat and tan-purple renal capsule. There was a well-defined cyst that measured 4.2 x 2.8 x 2.2 cm, centrally located in the upper to middle pole, extending into the renal cortex and medulla, grossly infiltrating the capsule and was tan-white in color. The cyst was filled with a scant amount of yellow-tinged fluid. The remaining parenchyma consisted of 0.8-cm-thick, pink-tan-colored cortex and 1.3-cm-thick, pale brown medulla with a well-defined corticomedullary junction. No infiltration of the vessels or ureter was noted. A microscopic examination of the tumor showed fibrous stroma and cystic areas. No significant atypia, mitoses or stromal was appreciated ruling out malignancy. Ureteral, vascular and peripheral soft tissue margins were free of tumor. Twelve excised peri-aortic lymph nodes were examined, ranging in greatest dimension from 0.4 to 1.2 cm with a tan-colored appearance. Only one of the 12 lymph nodes had an epithelial proliferation that favored to represent endosalpingiosis, which represents ectopic tubal epithelium and could be attributed to the fact that the patient had remotely underwent salpingectomy. Immunohistochemical staining performed on the renal specimen showed stromal components to have positivity for estrogen receptors (ER) and progesterone receptors (PR). PAX-8 was positive highlighting the epithelial elements. HMB-45 and Melan-A were negative. Immunohistochemical staining of the excised lymph nodes showed the glands within to be positive for PAX-8. P16 was negative and P53 showed a wild-type pattern. A definitive diagnosis of benign MEST was thus established post-operatively.

**Figure 3:**
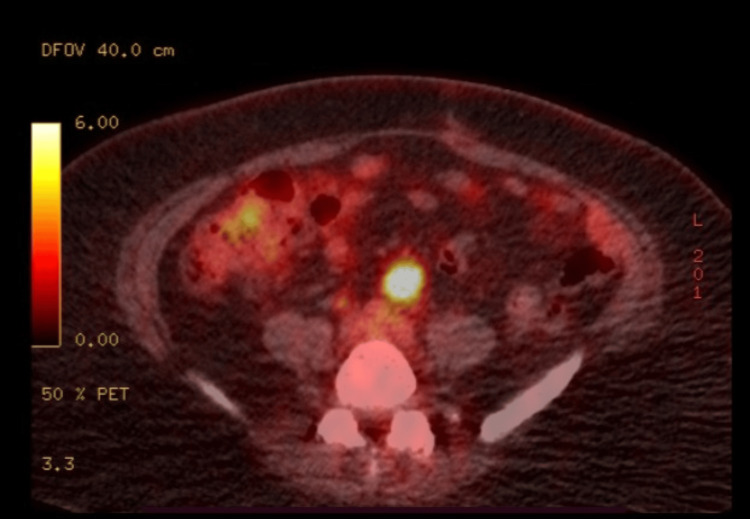
Hypermetabolic lymph node anterior to the aortic bifurcation as observed on the skull base to mid-thigh PET/CT scanning at the six-month follow-up PET/CT, positron emission tomography/computed tomography

At follow-up, six months post-surgery, the patient was asymptomatic and renal US confirmed post left nephrectomy status. However, a follow-up FDG-PET scan of the skull base to mid-thigh noted persistent nodal activity with a mesenteric node measuring 1.8 cm and demonstrating an SUV of 8.8 (Figure 3). No other adenopathy was noted. Considering the asymptomaticity and lack of other relevant imaging findings, the lymph node aligned more towards a benign pathology. Taking patient preferences into account, it was decided to follow her up with imaging studies every four to six months and proceed with invasive testing if symptomaticity recurred or the lesions displayed suspicious features upon imaging.

## Discussion

Mixed epithelial and stromal tumor was identified as a distinct entity by Michal and Syrucek in 1998 [2,6,7]. MESTs are rare tumors, accounting for just around 0.2% of renal neoplasms with sparse literature available on them. A MEST is a biphasic adult kidney tumor, comprising both epithelial and mesenchymal elements, featuring cysts, microcysts, and tubules with variable stromal components, requiring differentiation from other cystic tumors [8]. Close differential diagnoses include cystic nephroma, adult mesoblastic nephroma, leiomyosarcoma, renal synovial sarcoma, transitional cell carcinoma, and sarcomatoid renal cell carcinoma. Since MESTs and cystic nephromas share clinical, morphologic, histopathologic and immunohistochemical features, it has been suggested to merge these two diagnoses under "renal epithelial and stromal tumor (REST)" [7]. Our case typifies this uncommon neoplasm presentation, adding distinct features to the evolving understanding on MESTs.

MEST typically occurs in perimenopausal to older women, with the mean age at presentation being 45 years. Some patients present with urinary symptoms like hematuria, abdominal pain, palpable flank mass, renal calculi or recurrent urinary tract infections, but most are incidentally discovered on imaging. The recent literature highlights an upsurge in incidental findings due to increased use and advancement of radio-imaging modalities [9]. Our case mirrors this trend; our patient initially presented with hematuria and non-specific low-grade chronic pain in the left back.

On contrast-enhanced CT scan, MESTs usually reveal a solid or solid-cystic renal mass with mild to moderate, delayed enhancement of the solid component but no enhancement of the cystic component. FDG-PET scans usually show hypermetabolic activity. The conclusive diagnosis of MEST remains elusive through imaging since radiographic characteristics lack specificity, posing challenges for accurate preoperative diagnosis. Imaging in our patient showed features that have been previously ascribed to MESTs in the literature. The definitive diagnosis typically emerges postoperatively on histopathological analysis of the surgical specimen. The gross examination commonly shows a variably circumscribed, encapsulated, tan-yellow tumor, often with biphasic septated cystic growths, exhibiting diverse morphologies ranging from primarily cystic to predominantly solid [2,3], as is discerned in our report. MESTs have been reported to be noninvasive, well-circumscribed neoplasms, ranging in size from 2 to 24 cm [4,10]. The tumor reported in this patient measured 4.2 cm on gross examination. Microscopically, MEST exhibits a biphasic structure, comprising mesenchymal and epithelial elements. The mesenchymal component features spindle cells and smooth muscle cells, with sclerotic, fibroblastic or myofibroblastic features. The epithelial component is typically dispersed within the stroma and exhibits a spectrum from regular tubules to complex tubulopapillary structures, with or without cystic dilatation [11,12]. The absence of atypia, mitoses, pleomorphism, and necrotic changes supports a benign interpretation of the disease. The indolent behavior of our patient's MEST, evident from the minimal growth observed over a span of two years compared to initial CT scan findings, aligns with literature expectations.

A leading theory proposed to elucidate the pathogenesis of MEST implicates hormonal imbalance common to peri- and postmenopausal females explaining the gender predilection of MEST [9,10]. The mesenchymal component exhibits notable expression of estrogen and progesterone receptors, resembling ovarian stromal tissue. Recent findings by Turbiner et al. reported 62% ER expression and 85% PR expression in the stromal component of benign MESTs [7]. Our report corroborates the theory, especially considering our patient's body mass index of 38.4 kg/m^2^ and a past medical history of uterine fibroids and heavy menstrual bleeding requiring endometrial ablation, conditions strongly associated with altered hormonal milieu. Although the presence of estrogen and progesterone receptors might be indicative of MESTs, morphological features remain the primary diagnostic criteria. Distinct immunohistochemical staining patterns, chromosomal translocations and familial factors have also been implicated in the literature. On immunohistochemical profiling, the stromal component of MEST displays smooth muscle differentiation, typically staining positive for actin, desmin and H-caldesmon, and negative for melanocytic markers [1,3]. Positivity for calretinin, CD10, and inhibin has also frequently been noted and is indicative of steroidogenic origin [3,7]. In our patient, the tumor showed ER, PR and PAX-8 positivity and stained negative for HMB-45 and Melan-A, conforming to the staining patterns postulated in the literature. Surgical intervention is the typical treatment approach, leading to postoperative pathological diagnosis [13]. No guidelines exist on the follow-up surveillance protocol and challenges arise due to the absence of clear clinical or radiological findings [3].

The majority of studies in the literature report benign tumors with or without local invasion and without recurrence. Prognosis is reported to be excellent in most cases after undergoing surgical resection. Albeit rare, there are two reported future complications: malignant transformation and local recurrence. Malignant variants present with aggressively enlarging tumors and may display rhabdoid, rhabdomyosarcomatous, and chondrosarcomatous elements on histological evaluation [14]. Sarcoma has been reported to be the commonest variant [14]. The diagnosis of malignant MESTs rest on identifying characteristic epithelial elements with associated stromal cuffing [15]. Also, rare instances of local and distant recurrences of MEST after both partial and radical nephrectomies have been reported [16,17]. In our case, a follow-up FDG-PET scan at six months after surgery noted persistent nodal activity; however, the patient was asymptomatic and doing well. Clinical significance of such lymph node appearances on the FDG-PET scan is, at present, unknown. However, long-term follow-up is advised in view of known future complications.

## Conclusions

Mixed epithelial and stromal tumors of the kidney are exceptionally rare, benign renal neoplasms with a predilection for peri/postmenopausal women that should be considered in patients presenting with non-specific abdominal and/or genitourinary symptoms, probably stemming from hormonal imbalance. Diagnosis relies heavily on histopathology, and caution is advised against limited tumor sampling techniques. Outcomes are generally favorable with conservative surgery. However, a meticulous long-term follow-up is recommended considering the rare chances of local recurrence and malignant transformation.
